# Exposure to hyperbaric O_2_ levels leads to blood-brain barrier breakdown in rodents

**DOI:** 10.1186/s12987-024-00543-7

**Published:** 2024-05-16

**Authors:** Yehuda M. Danino, Ricarina Rabinovitz, Inbar Kirshenboim, Eilam Palzur, Chaim G. Pick, Itamar Ish-Shalom, Yana Golovkin, Yehuda Arieli

**Affiliations:** 1 Israel Naval Medical Institute, P.O. Box 8040, Haifa, 31080 Israel; 2 Research Institute of Galilee Medical Center, P.O.Box 21, Nahariya, 22100 Israel; 3 Department of Anatomy and Anthropology, Sackler School of Medicine, Tel-Aviv University, Tel-Aviv, Israel; 4 Sagol School of Neuroscience, Tel-Aviv University, Tel-Aviv, Israel; 5 Dr. Miriam and Sheldon G. Adelson Chair and Center for the Biology of Addictive Diseases, Tel-Aviv University, Tel-Aviv, Israel

**Keywords:** BBB Permeability, 100% oxygen, Atmospheric absolute, Hyperbaric chamber, CNS oxygen toxicity, CNS-OT, Rats, Mice

## Abstract

**Introduction:**

Hyperbaric oxygen has been used as a medical treatment tool in hyperbaric chambers and is an integral part of professional and combat divers’ activity. In extreme cases, exposure to hyperbaric oxygen can develop central nervous system oxygen toxicity (CNS-OT), which leads to seizures and eventually death. CNS-OT is caused by neuronal hyperactivity due to high oxygen levels, potentially damaging brain cells including the blood-brain barrier (BBB). However, the effect of hyperbaric oxygen levels on the healthy BBB has not been characterized directly yet.

**Methods:**

Six or three different groups of ~ eight rats or mice, respectively, were exposed to increasing levels of partial pressure of oxygen (0.21 to 5 ATA) in a hyperbaric chamber, followed by MRI scanning with gadolinium. Statistical significance (*adjusted p-value ≤ 0.05*) was assessed using linear regression and ordinary one-way (rats) or two-way (mice) ANOVA with correction of multiple comparison tests. In rats, the effect of 100% oxygen at 5 ATA was independently validated using FITC-Dextran (5 kDa). Statistical significance (*p-value ≤ 0.05*) was assessed using Welch’s t-test and effect size was calculated by Cohen’s D.

**Results:**

In rats, analyzed MRI scans showed a significant trend of increase in the % gadolinium in brain tissues as a result of hyperbaric oxygen pressures (*p-value = 0.0079*). The most significant increase was measured at 4 ATA compared to air (*adjusted p-value = 0.0461*). Significant increased FITC-Dextran levels were measured in the rats’ brains under 100% oxygen at 5 ATA *versus* air (*p-value = 0.0327;* Effect size = 2.0). In mice, a significant increase in gadolinium penetration into the hippocampus and frontal cortex was measured over time (*adjusted p-value < 0.05*) under 100% oxygen at 3 and 5 ATA *versus* air, and between the treatments (*adjusted p-value < 0.0001*).

**Conclusions:**

The BBB is increasingly disrupted due to higher levels of hyperbaric oxygen in rodents, indicating a direct relation between hyperbaric oxygen and BBB dysregulation for the first time. We suggest considering this risk in different diving activities, and protocols using a hyperbaric chamber. On the other hand, this study highlights the potential therapeutic usage of hyperbaric oxygen for controlled drug delivery through the BBB into brain tissues in different brain-related diseases.

## Introduction

Recent years have seen continuous growth and expansion in the use of the hyperbaric chamber as treatment of an increasing number of clinical indications. This has led to greater numbers of patients being exposed to mildly elevated partial pressures of oxygen (ppO_2_) on a routine, daily basis. Healthy individuals, such as recreational, professional, and combat divers, who use oxygen-enriched breathing gas mixtures, face a similar physiological challenge. However, we have scant knowledge regarding the effects of routine exposure to hyperbaric oxygen (HBO) on healthy persons [1–3]. Treatment with normobaric oxygen seems to provide protection against a variety of brain injuries, mainly via the improvement of blood-brain barrier (BBB) permeability [4–7]. Although it is widely accepted that the mildly elevated ppO_2_ to which HBO patients and divers are exposed is sub-toxic or harmless [8], other studies dispute this assumption. Patients in the hyperbaric chamber have been known to develop seizures [9], sometimes accompanied by cardiogenic pulmonary edema [10–12]. Reports of the administration of HBO in patients diagnosed with cerebral stroke or ischemia-reperfusion injury have provided conflicting results [13, 14]. Exposure to a high ppO_2_ may lead to increased production of reactive oxygen species (ROS), which are known to be anxiety-inducing agents [15]. Moreover, Domachevsky et al. [16, 17] administered a higher ppO_2_ at 4–5 atmospheres absolute (ATA) to mice, until the onset of central nervous system oxygen toxicity convulsions. They noted activation of an apoptosis cascade in the hippocampus, delayed injury of the BBB, and transient impairment of performance on behavioral tests. Additional studies provide evidence for the claim that impaired BBB integrity is one of the major suspected effects of injury due to increased levels of ppO_2_ [18, 19]. However, the way by which hyperbaric oxygen levels affect BBB permeabilization is not fully understood.

Therefore, the purpose of the present investigation is to study the effect of different levels of ppO_2_ on BBB integrity in healthy rodents. Here, we exposed health rats and mice to elevated ppO_2_ levels exceeding normobaric pressure, which is defined as 21% O_2_ at 1 ATA. Through MRI scans, we illustrate that exposure to increased ppO_2_ levels, even at sub-toxic pressures such as 100% oxygen at 5 ATA, induces BBB breakdown. Orthogonally, a significant fluorescent signal was detected in rat brains after exposure to 5 ATA, compared to those exposed to a normobaric environment, supporting the MRI results. These findings represent a direct relation between hyperbaric oxygen and BBB dysregulation for the first time. This implies that elevated ppO_2_, even at sub-toxic levels, may cause impairment of the BBB. This study paves the way for the development of oxygen-based drug delivery across the BBB into the CNS, and safer hyperbaric oxygen therapy protocols.

## Materials

### Animals

Sprague-Dawley male rats weighing 200–218 g and ICR male mice weighing 25–30 g, were purchased from Envigo RMS, Israel.

Animals were kept at room temperature in a 12/12 light/dark cycle, in standard plastic cages. Water and food were freely accessible, and the layer of sawdust on the cage floor was replaced twice a week, similarly for each cage. On their arrival at the laboratory, animals were given a week to recover from transportation and for acclimatization to the new location. Two days before the experiment, all the cages were transferred to the experimental room and were placed for a time inside the pressure chamber, to enable the animals to become accustomed to the new environment and to reduce anxiety.

### HBO exposure protocols

**Fig. 1:**
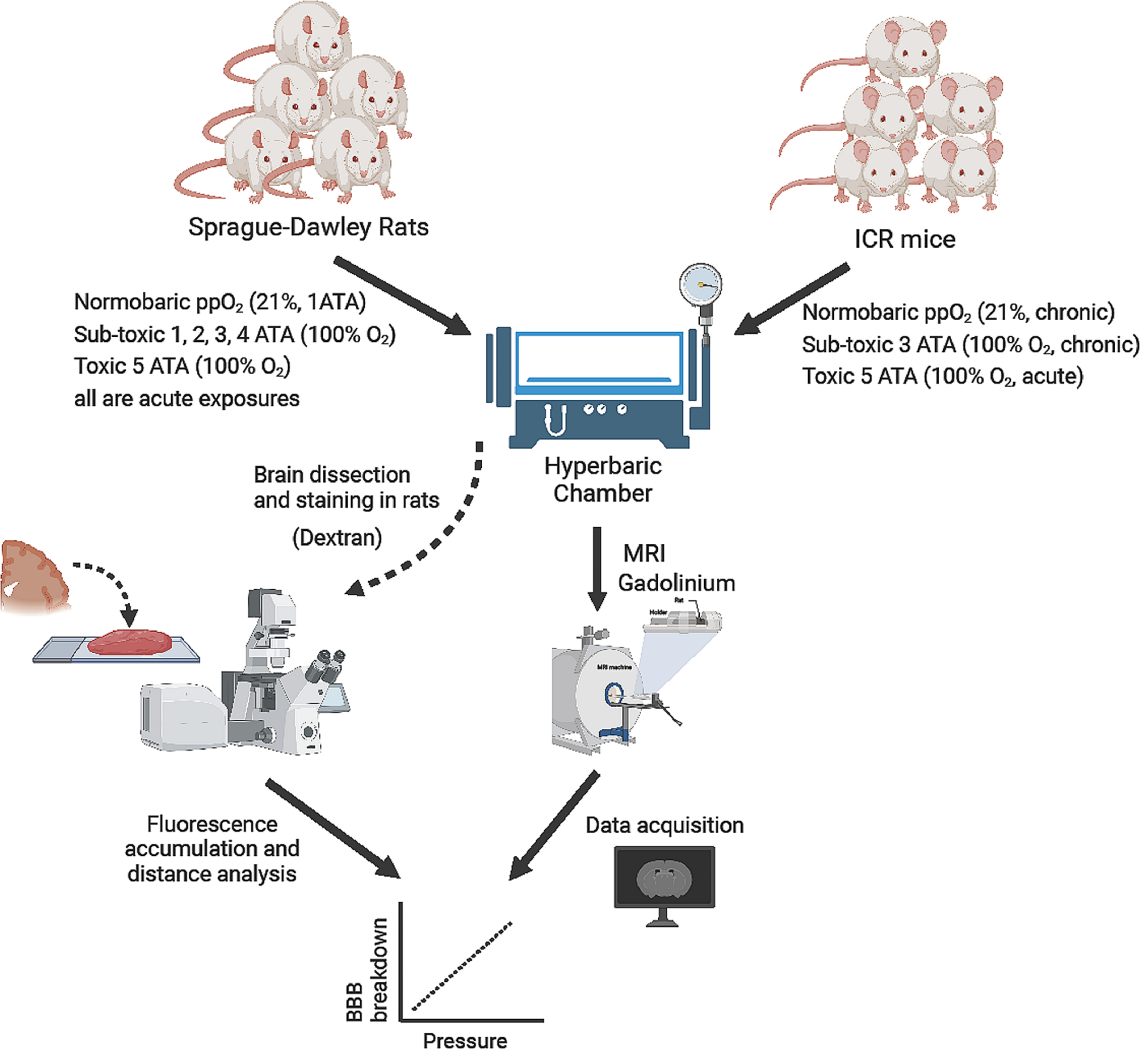
Study design. Sprague-Dawley male rats or ICR male mice were exposed to hyperbaric oxygen by using a hyperbaric chamber in different protocols containing normobaric, sub-toxic, and toxic O_2_ pressures. Then, the animals were injected with gadolinium followed by MRI scans. The MRI data (gadolinium distribution) were analyzed. Equivalent groups of the rats were concurrently injected with dextran and the rats’ brains were dissected for staining and imaging by fluorescent confocal microscopy. Illustration was generated through Biorender.com

To investigate hyperbaric oxygen’s impact on the rat brain’s blood-brain barrier (BBB), 48 rats were randomly assigned to six groups, each comprising eight rats. These groups were exposed to different oxygen levels, of either 21% or 100% oxygen, across five pressure levels (1–5 ATA). The oxygen partial pressure (ppO_2_) is the primary physiological measure of oxygen perception. Considering both oxygen levels and environmental pressure enables us to determine the ppO_2_, which is defined by the next equation: ppO_2_= [pressure] X [% O_2_ in the mixture]. Thus, the ppO_2_ value expresses both the surrounding pressure and the levels of O_2_ that the animal is exposed to. The control group was subjected to normbaric air, which is composed of 21% oxygen at 1 ATA, i.e. a ppO_2_ of 0.21 ATA. These conditions for the control group were chosen based on previously published papers [17, 20, 21]. The other groups were exposed to 100% oxygen at one of five pressures (1–5 ATA) in the hyperbaric chamber **(**Fig. 1**)**, resulting in ppO_2_ levels ranging from 1 to 5 ATA. To understand how the BBB in different zones in mice brains are affected by hyperbaric oxygen, mice were randomly divided into three experimental groups. The first group served as a control group, in which animals were exposed to normobaric air (0.21 ppO_2_ at 1 ATA). To simulate chronic sub-toxic exposure, mice in the second group were subjected to three sessions of pure oxygen at 3 ATA for 60 min, once every other day, 2 animals at a time. The third group was the toxic group, in which animals were exposed one at a time to a single session of pure oxygen at 5 ATA until the appearance of convulsions, in an attempt to understand the effect of HBO plus convulsions on the BBB. Animals in each experimental group underwent MRI scanning (*n* = 8 from each group), with gadolinium (GD) as a marker.

For both animal models, in the case of seizures, the duration of the exposures was between 7 and 16 min. We compressed the animals in a small pressure chamber 31.5 cm in diameter and 23 cm high, provided with a glass window 12 cm in diameter on the top cover. To enable continuous observation of mice inside the chamber, we placed them in a smaller open plastic box 8.5 cm in breadth, 12.5 cm long, and 12.5 cm high, which was positioned under the glass window.

### MRI acquisition

MRI was performed with a 7T MRI scanner (Bruker, Karlsruhe, Germany), having a 30 cm bore and a gradient strength of up to 660 mT/m, using a four-channel quadrature head coil. Mice and rats were anesthetized with 1‒2% isoflurane and oxygen, body temperature was maintained at 37 °C, and their breathing rate was monitored by means of a breathing sensor. A dynamic contrast enhancement (DCE) protocol was employed, with the following parameters: T1 weighted (FLASH) sequence was performed with three repetitions as a baseline, and 30 repetitions following intraperitoneal injection of 0.15 ml, 0.5 M gadolinium-DTPA (GD-DTPA). Sequence parameters were TE = 2.5 ms; TR = 150 ms; 4 averages; and scan time of 30 s per repetition. Geometrical parameters were: 12 coronal slices of 0.6 mm thickness (brain volume) and in-plane resolution of 0.12 × 0.12 mm^2^ (matrix size 160 × 128 mm and FOV 19.2 × 15.36 mm).

### DCE analysis

For rats, we conducted a whole-brain analysis. For mice, we focused on two specific areas: the hippocampus and the frontal cortex. The DCE analysis comprised the following procedures: Motion correction was performed to correct for motion artifacts using SPM software (version 12, UCL, London, UK); GD-DTPA enhancement maps (post-injection signal intensity minus pre-injection signal intensity) were calculated in a voxel-based manner for each repetition following GD enhancement; Region of interest (ROI) analysis was performed by outlining manually on the baseline image. Outlined regions included in rats’ whole brain slices in similar depth. In mice, it included the hippocampus and frontal lobe in both left and right hemispheres; The percent of GD enhancement was calculated in each ROI for positive enhancement voxels and for voxels larger than 10% enhancement.

### Fluorescent imaging

In rats, we validated the MRI study with an orthogonal fluorescence-based approach independently using FITC-Dextran as a marker. Rat maintenance was as described above. Rats were introduced to the pressure chamber for acclimatization before a single exposure session of 45 min at 4 ATA of pure oxygen. Prior to the experimental exposure, 1 ml of FITC-Dextran (5 kDa) rats was injected via the tail vein, under anesthesia with isoflurane. Immediately after descending, rats were anesthetized with equithesin (4 ml/kg), and blood was drained out via the right ventricle by injecting cold saline into the left ventricle, followed by cold paraformaldehyde (4%). The brain was immediately removed and prepared for fluorescence microscopy observation according to a protocol described by Natarajan et al. [22]. Briefly, brains were immersed in paraformaldehyde (4%) for 48 h. Brains were then immersed in glycerol 10% followed by 20% and stored at -80 °C until sliced in a cryostat. Twenty-two to twenty-five images from 5 different animals per group were collected using a fluorescent confocal microscope (Zeiss), x10 magnification. The images were quantified in a single-blinded manner. FITC-Dextran fluorescence values were evaluated as mean gray values (normalized to the area of the image) using ImageJ software (NIH) [23]. For each group, the values were averaged and the results are shown as mean ± SE.

### Statistical analysis

All results are presented as mean (SE). Data from MRI analysis were subjected to one-way ANOVA with Dunnett’s multiple compression test and linear regression (in rats) or two-way ANOVA with Dunnett’s or Tukey’s multiple comparison test (in mice). For fluorescent analysis, an unpaired t-test with Welch’s correction was done. The statistical analyses and plots were obtained by using GraphPad Prism 9 software.

## Results

### Hyperbaric pressure of oxygen increases the BBB permeability in rats

**Fig. 2:**
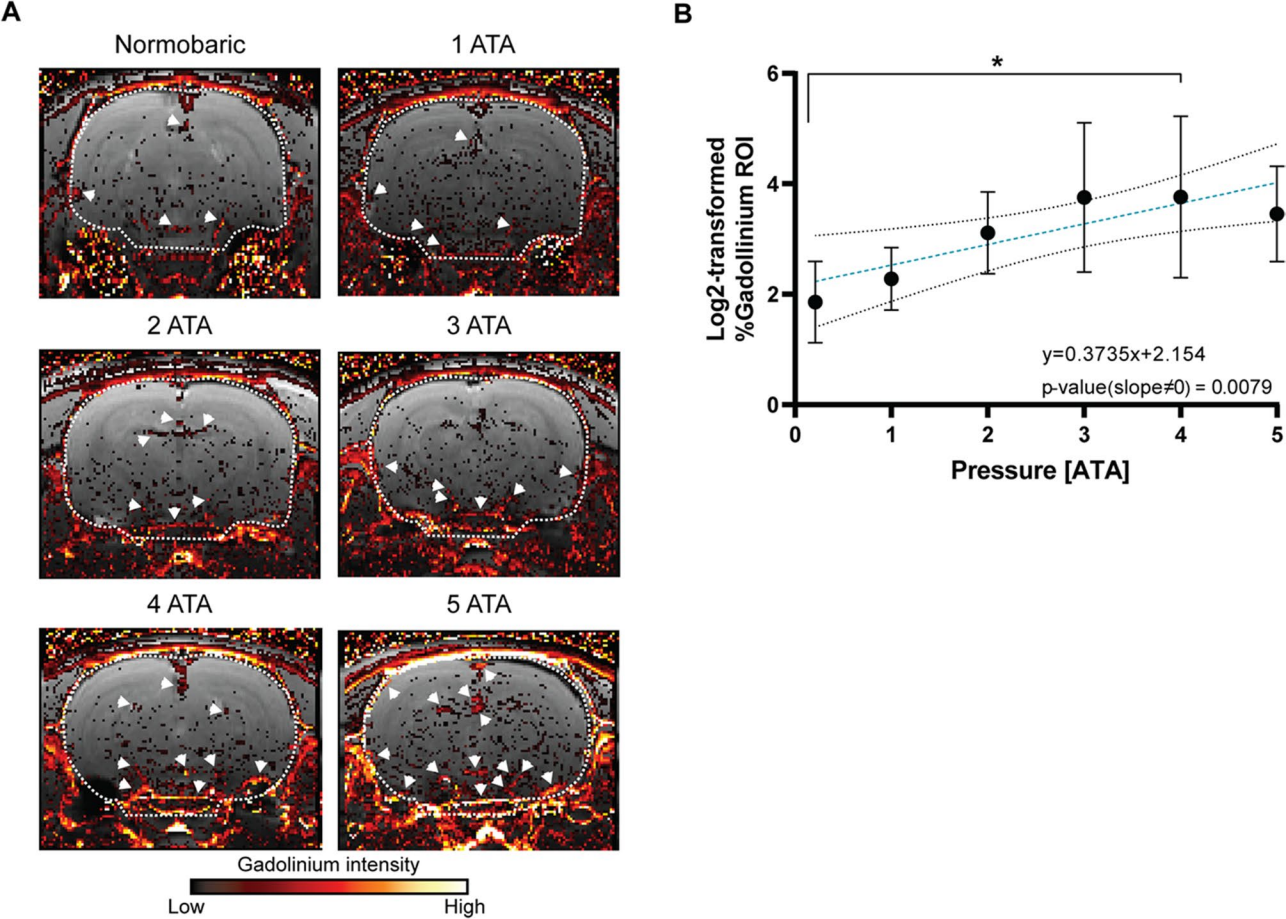
BBB is disrupted as a result of 100% ppO_2_ in rats. (**A**) Representative samples of consecutive whole-brain MRI slices from different experimental rat groups (exposed to different pressures) were taken over a period of 30 min post-gadolinium-DTPA (GD) administration. The fire-colored areas indicate a percent change in GD enhancement (see scale bar), overlaid on the grayscale sMRI T1 weighted image before GD injection. The ROI in each representative image is denoted by a white dashed line. White arrowheads indicate GD leakage events in the representative MRI slice images. (**B**) A scatter plot shows the average quantified percentages of the GD area (ROI; region of interest) in each group of rats per condition 30 min post-treatment (different O_2_ pressure). The equation is noted. * *p-value < 0.05*, one-way ANOVA with Dunnett’s multiple compression test. Linear regression was performed to evaluate the change between the different treatments compared to normobaric air (0.21 ATA ppO_2_), which is represented by a linear equation containing a different slope value from zero, *p-value = 0.0079*. The data is mean ± SE. ATA; Atmosphere absolute units

BBB integrity is suggested to be injured by increased levels of ppO_2_ [18, 19]. To test this hypothesis, we scanned whole brains of rats 30 min after acute exposure to different pressures ranging from normobaric air to the toxic state of 100% oxygen at 5 ATA using MRI (Figures 1 and 2A). More events of gadolinium (GD) leakage were observed in MRI scans of rat brains subjected to hyperbaric pressure (Fig. 2A; white arrowheads). Upon further analysis, the scans revealed a significant increase in the percentage of gadolinium leakage within the ROI, which includes the entire brain slice area (white dashed line; whole brain), with an increase in oxygen pressure (5 ATA compared to air), represented by linear regression (*p-value* *= 0.0079*). Interestingly, the GD penetration into the brain at 4 ATA was the most significant compared to air condition (Ordinary one-way ANOVA with Dunnett’s multiple comparison tests, *adjusted p-value = 0.0461*; Fig. 2B).

**Fig. 3:**
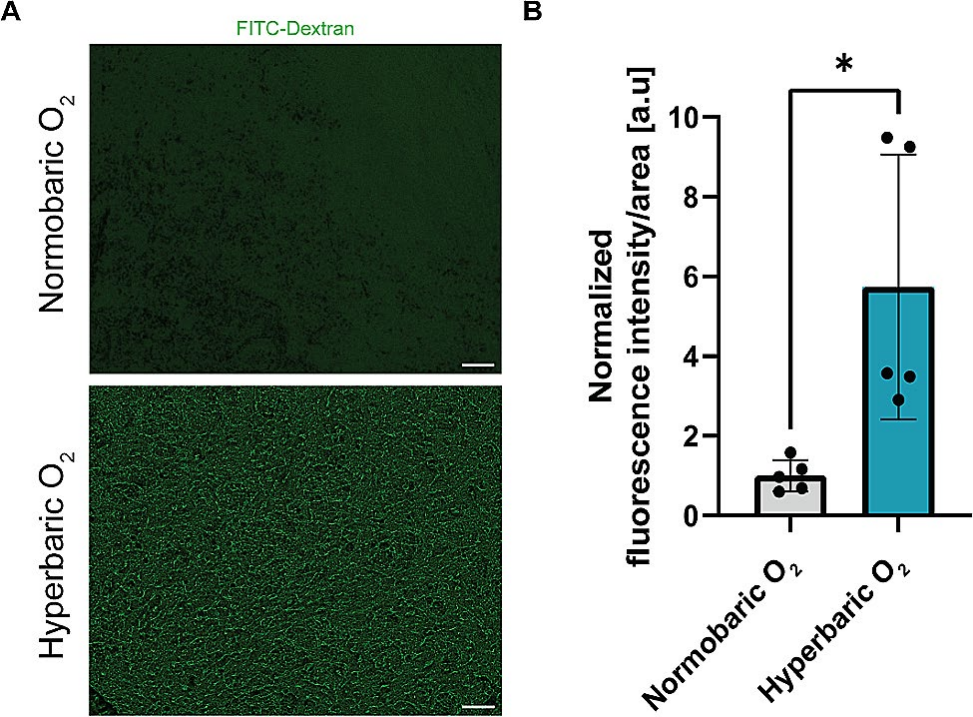
Hyperbaric oxygen leads to BBB disruption compared to normobaric oxygen in rats. **(A)** Representative fluorescent micrographs of rat brain slices under normobaric O_2_ (upper image) or hyperbaric O_2_ (5 ATA; lower image) indicating FITC-Dextran fluorescent signal (green). Magnification X10; scale bar = 100 μm. **(B)** Bar plot displays the difference in normalized dextran fluorescence in rat brains after normobaric air exposure compared with hyperbaric exposure representing the BBB disruption. Anesthetized (Equithesin 4 ml/Kg) rats were injected via the tail vein with FITC dextran, 45 min before treatment in the hyperbaric chamber. Following exposure to normobaric air or 100% O_2_ at 5 ATA, rats were immediately sacrificed and the brains were dissected and fixed for imaging in a fluorescent confocal microscope. The fluorescent signal of the dextran was quantified and normalized to the area using slices of 10 μm in thickness. * *p-value < 0.05*, Unpaired t-test with Welch’s correction. The data is mean ± SE. ATA; Atmosphere absolute units

Moreover, an orthogonal approach that measures the fluorescence signal of FITC-Dextran (5 kDa) in the rats’ brains showed significantly elevated levels of the signal after exposure to pure oxygen at 5 ATA compared to air (Fig. 3A), supporting the MRI results **(**Welch’s t-test, *p-value = 0.0327*; Fig. 3B). Altogether, these results suggest that the BBB is increasingly disrupted as the 100% oxygen pressure increases, specifically in the toxic pressure range of 4–5 ATA.

### Hyperbaric oxygen enhances BBB permeability in mice

**Fig. 4:**
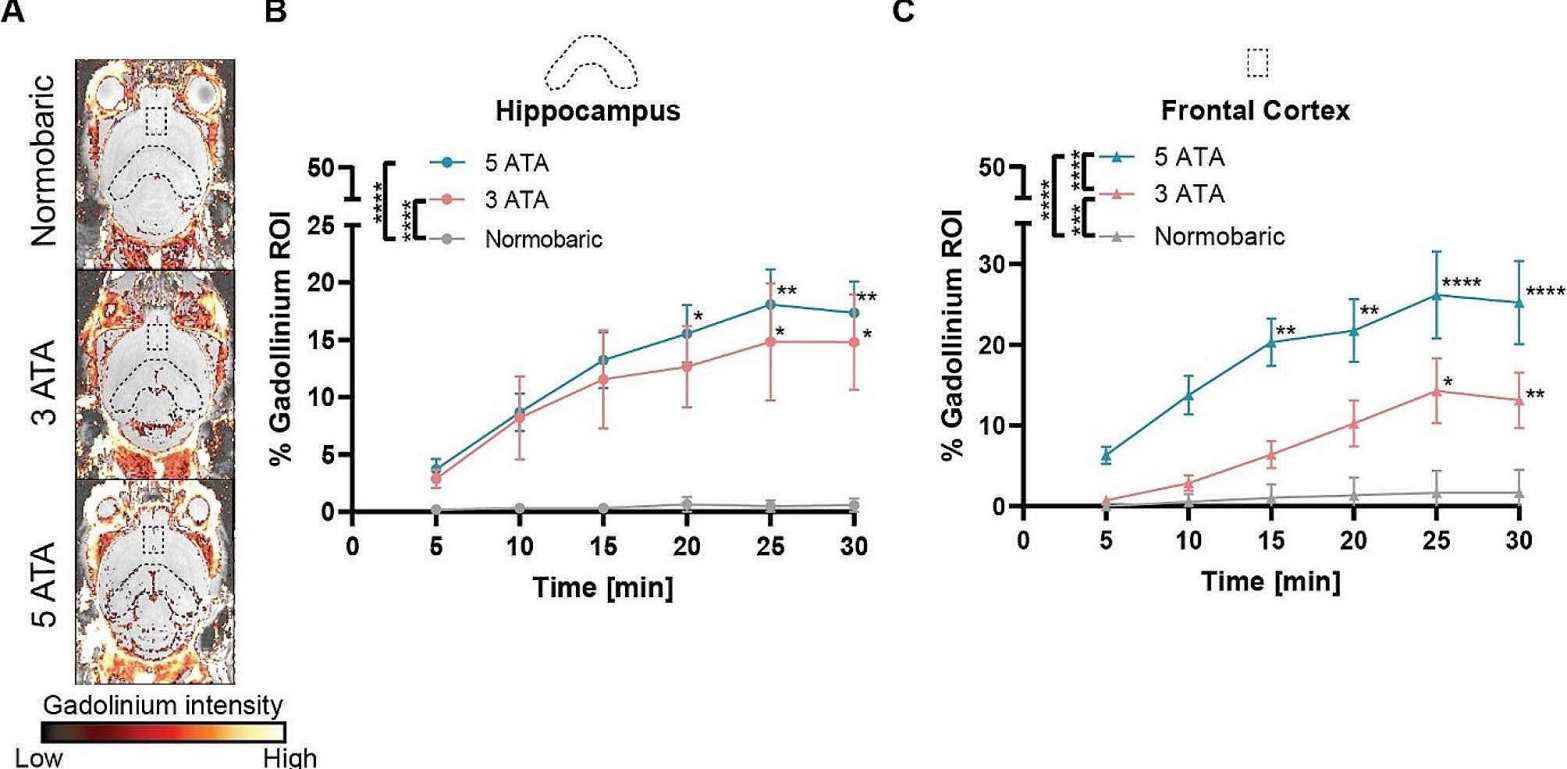
BBB is disrupted as a result of high 100% ppO_2_ in mice. **(A)** Representative samples of consecutive whole-brain MRI slices from different experimental mice groups (exposed to different pressures and exposure protocols) were taken over a period of 30 min post-gadolinium-DTPA (GD) administration. The fire-colored areas indicate a percent change in GD enhancement (see scale bar), overlaid on the grayscale sMRI T1 weighted image before GD injection. The ROI in each representative image is denoted by a black dashed line. In each image, the upper square-shaped ROI indicates the frontal cortex; the lower curved oval-shaped ROI indicates the hippocampus. **(B, C)** Scatter plots show the average quantified percentages of the GD area (ROI; region of interest) in each group of mice per condition during the 30 min following treatment (chronic normobaric air or 100% oxygen 3 ATA or acute 100% oxygen 5 ATA) at 5-minute intervals, in the **(B)** Hippocampus or **(C)** Frontal cortex. * *p-value < 0.05*, ** *p-value < 0.01*, *** *p-value < 0.001*, **** *p-value < 0.0001*; Two-way ANOVA with Tukey’s or Dunnett’s multiple compression tests were performed for comparison between groups or time points within each group, in relation to the first time point, respectively. The data is mean ± SE. ATA; Atmosphere absolute units

Next, to further establish and expand the hypothesis according to which 100% oxygen at high pressures leads to BBB disruption, we used mice as an independent and non-related rodent model to rats. We chronically or acutely exposed the mice to sub-toxic pressures (normobaric air and 100% oxygen at 3 ATA) or toxic state (100% oxygen at 5 ATA), respectively, and then scanned their brains 5, 10, 15, 20, 25, and 30 min after the exposure using MRI **(**see Materials, Figures 1 and 4A**)**. Based on the findings in rats (Fig. 2), the MRI scans conducted on mice focused on the hippocampus (curved oval-shaped ROI; Fig. 4A) and frontal cortex (square-shaped ROI; Fig. 4A) areas. Both areas are known to play major parts in the induction and propagation of seizures leading to convulsions. The analysis of MRI data revealed a significant increase in the concentration of gadolinium in brain tissues over time (Two-way ANOVA with Dunnett’s multiple compression tests, *adjusted p-value < 0.05*; Fig. 4B, C). In addition, a significant increase in the presence of gadolinium in the brain tissues was also observed between the treatments (Two-way ANOVA with Tukey’s multiple correction tests, *adjusted p-value < 0.0001*; Fig. 4B, C). In both brain regions, the peak of the gadolinium levels was seen 25 min after administration in both 3 ATA and 5 ATA conditions (Fig. 4B, C). Notably, significantly increased levels of gadolinium were found with increasing levels of ppO_2_ earlier under exposure to 5 ATA compared to 3 ATA, relative to the first measurement **(**Fig. 4B, C), expressing the extreme effect of the toxic state (5 ATA). Moreover, the 5 ATA group demonstrated a greater increase in gadolinium levels compared to the normobaric and 3 ATA groups in the frontal cortex (Fig. 4C). However, in the hippocampus, non-significant differences between the gadolinium levels between 5 ATA versus 3 ATA groups were shown **(**Fig. 4B).

Together with the data obtained from the rat model, these observations, which display increased gadolinium levels in the mouse brain as a result of increased pressures of 100% oxygen in time and in different protocol types, support the suggestion that the BBB permeability is enhanced in relation to higher oxygen pressure.

## Discussion

In the present study, we investigated the effect of different HBO exposure protocols on the brains of two animal models: the rat and the mouse. The MRI study of both rodent brain samples revealed a significant increase in GD-DTPA dispersal in relation to the increased pressure of 100% oxygen, in acute or chronic exposures and under sub-toxic or toxic 5 ATA pressures (Figs. 2 and 4). Although the use of MRI does not enable us to determine whether the increased dispersal of gadolinium in brain tissue is the result of vasodilation or significant BBB disruption, the known vasoconstrictive effect of O_2_ allows us to reasonably assume that the observed MRI results were not due to vasodilation. Furthermore, we found more gadolinium following the different HBO exposure protocols and pressures compared to the control groups, this would rule out vasodilation as a possible mechanism. Moreover, this conclusion is strengthened by results from the fluorescence imaging experiments in rats showing an increased FITC-dextran signal in the brain under 100% oxygen at 5 ATA compared to the condition of normobaric air (Fig. 3). We therefore suggest this is more likely to be the result of BBB disruption.

Comparison between the effect of high partial pressure of oxygen at different conditions on the hippocampus and frontal cortex, in mice, demonstrated that the increase in GD-DTPA in these brain regions seems to be ppO_2_-dependent. Regarding this topic, Mattos et al. [18] concluded that during isocapnic hyperoxia, ROS play a role in the regulation of cerebral blood flow and metabolism, without evidence of BBB disruption, whereas Liu et al. [24] found normobaric hyperoxia to attenuate early BBB disruption. A previous study conducted in our laboratories demonstrated BBB disruption following seizures induced by HBO exposure [17]. According to the findings of the present study, HBO exposure (both toxic and sub-toxic) disrupts the BBB in the two brain regions under investigation. Despite that, a more detailed study should be performed to investigate the response of the BBB to elevated ppO_2_ in the hippocampus and frontal cortex, which are different brain regions with distinct roles. Moreover, characterizing the mechanism (e.g. paracellular or transcellular) underlying the BBB breakdown is an important subject to be addressed in the future. In addition, the present study needs to be extended, with follow-up studies in humans to pave the way to develop low-risk treatment protocols in hyperbaric chambers and oxygen-based strategies for drug delivery into the CNS through the BBB for different CNS-associated diseases including brain cancers, neurodevelopmental and neurodegenerative disorders.

## Conclusions

Based on the results above in two independent animal models (rats and mice), we can conclude that there is a direct relation between hyperbaric oxygen and BBB breakdown: higher hyperbaric oxygen levels lead to increased BBB dysregulation. To our knowledge, this relation is reported here as a direct connection for the first time. This study has two very different implications: On one hand, the risk of BBB disruption under hyperbaric pressure needs to be considered in diving activities and hyperbaric oxygen therapy protocols. On the other hand, this study paves the way for potential therapeutic usage of hyperbaric oxygen for controlled drug delivery through the disrupted BBB into brain tissues in cases of brain cancers, neurodevelopmental disorders, and neurodegenerative diseases.

## Data Availability

No datasets were generated or analysed during the current study.

## Abbreviations

CNS-OT: Central nervous system oxygen toxicity
ppO_2_: Partial pressure of oxygen
HBO: Hyperbaric oxygen
ATA: Atmosphere absolute
BBB: Blood-brain barrier
ROI: Region of interest
GD: Gadolinium
ROS: Reactive oxygen species
MRI: Magnet resonance imaging
DCE: Dynamic contrast enhancement

## References

[1] Liang J, Qi Z, Liu W, Wang P, Shi W, Dong W (2015). Normobaric hyperoxia slows blood-brain barrier damage and expands the therapeutic time window for tissue-type plasminogen activator treatment in cerebral ischemia. Stroke.

[2] Liu W, Chen Q, Liu J, Liu KJ (2011). Normobaric hyperoxia protects the blood brain barrier through inhibiting Nox2 containing NADPH oxidase in ischemic stroke. Med Gas Res.

[3] Michalski D, Hobohm C, Weise C, Pelz J, Heindl M, Kamprad M (2012). Interrelations between blood-brain barrier permeability and matrix metalloproteinases are differently affected by tissue plasminogen activator and hyperoxia in a rat model of embolic stroke. Med Gas Res.

[4] Chen Z, Ding Y, Ji X, Meng R (2020). Advances in Normobaric Hyperoxia Brain Protection in Experimental Stroke. Front Neurol.

[5] Zhou W, Marinescu M, Veltkamp R (2015). Only very early oxygen therapy attenuates posthemorrhagic edema formation and blood-brain barrier disruption in murine intracerebral hemorrhage. Neurocrit Care.

[6] Hu W, Li W, Mangal R, Jia M, Ji X, Ding Y (2023). Normobaric Hyperoxia (NBHO): an adjunctive therapy to Cerebrovascular recanalization in ischemic stroke. Aging Dis.

[7] Shi S, Qi Z, Ma Q, Pan R, Timmins GS, Zhao Y (2017). Normobaric Hyperoxia reduces blood occludin fragments in rats and patients with Acute ischemic stroke. Stroke.

[8] Poff AM, Kernagis D, D’Agostino DP (2016). Hyperbaric environment: Oxygen and Cellular damage versus Protection. Compr Physiol.

[9] Warchol JM, Cooper JS, Diesing TS (2017). Hyperbaric oxygen-associated seizure leading to stroke. Diving Hyperb Med.

[10] Cho K, Minami T, Okuno Y, Kakuda Y, Tsutsumi T, Kogame T (2018). Convulsive seizure and pulmonary edema during hyperbaric oxygen therapy:a case report. J Med Invest.

[11] Pulmonary Edema Associated With Hyperbaric Oxygen Therapy (2001). Chest.

[12] Obiagwu C, Paul V, Chadha S, Hollander G, Shani J (2015). Acute pulmonary edema secondary to hyperbaric oxygen therapy. Oxf Med Case Rep.

[13] Li HZ, Chen JF, Liu M, Shen J (2018). Effect of hyperbaric oxygen on the permeability of the blood-brain barrier in rats with global cerebral ischemia/reperfusion injury. Biomed Pharmacother.

[14] Veltkamp R, Siebing DA, Sun L, Heiland S, Bieber K, Marti HH (2005). Hyperbaric oxygen reduces blood-brain barrier damage and edema after transient focal cerebral ischemia. Stroke.

[15] Olsen RHJ, Johnson LA, Zuloaga DG, Limoli CL, Raber J (2013). Enhanced hippocampus-dependent memory and reduced anxiety in mice over-expressing human catalase in mitochondria. J Neurochem.

[16] Domachevsky L, Pick CG, Arieli Y, Krinsky N, Abramovich A, Eynan M (2012). Do hyperbaric oxygen-induced seizures cause brain damage?. Epilepsy Res.

[17] Domachevsky L, Pick CG, Peled N, Gomori JM, Abramovich A, Tempel-Brami C (2013). MRI findings after hyperbaric oxygen-induced seizures. Epilepsy Res.

[18] Mattos JD, Campos MO, Rocha MP, Mansur DE, Rocha HNM, Garcia VP (2019). Human brain blood flow and metabolism during isocapnic hyperoxia: the role of reactive oxygen species. J Physiol.

[19] Noseworthy MD, Bray TM (2000). Zinc deficiency exacerbates loss in blood-brain barrier integrity induced by hyperoxia measured by dynamic MRI. Proc Soc Exp Biol Med.

[20] Henninger N, Küppers-Tiedt L, Sicard KM, Günther A, Schneider D, Schwab S (2006). Neuroprotective effect of hyperbaric oxygen therapy monitored by MR-imaging after embolic stroke in rats. Exp Neurol.

[21] Eynan M, Krinsky N, Biram A, Arieli Y, Arieli R (2014). A comparison of factors involved in the development of central nervous system and pulmonary oxygen toxicity in the rat. Brain Res.

[22] Natarajan R, Northrop N, Yamamoto B (2017). Fluorescein Isothiocyanate (FITC)-Dextran Extravasation as a measure of blood-brain barrier permeability. Curr Protoc Neurosci.

[23] Schindelin J, Arganda-Carreras I, Frise E, Kaynig V, Longair M, Pietzsch T (2012). Fiji: an open-source platform for biological-image analysis. Nat Methods.

[24] Liu W, Hendren J, Qin XJ, Shen J, Liu KJ (2009). Normobaric hyperoxia attenuates early blood-brain barrier disruption by inhibiting MMP-9-mediated occludin degradation in focal cerebral ischemia. J Neurochem.

[25] National Research Council, Division on Earth and Life Studies. Institute for Laboratory Animal Research, Committee for the update of the guide for the Care and Use of Laboratory animals. Guide for the Care and Use of Laboratory animals: Eighth Edition. National Academies; 2011. p. 246.

